# mRNA-Based Vaccines and Therapeutics for COVID-19 and Future Pandemics

**DOI:** 10.3390/vaccines10122150

**Published:** 2022-12-15

**Authors:** Vivek P. Chavda, Shailvi Soni, Lalitkumar K. Vora, Shruti Soni, Avinash Khadela, Jinal Ajabiya

**Affiliations:** 1 Department of Pharmaceutics and Pharmaceutical Technology, LM College of Pharmacy, Ahmedabad 380009, Gujarat, India; 2 Massachussets College of Pharmacy and Health Science, 19 Foster Street, Worcester, MA 01608, USA; 3 School of Pharmacy, Queen’s University Belfast, 97 Lisburn Road, Belfast BT9 7BL, UK; 4 PharmD Section, LM College of Pharmacy, Ahmedabad 380009, Gujarat, India; 5 Department of Pharmacology, LM College of Pharmacy, Ahmedabad 380009, Gujarat, India; 6 Department of Pharmaceutics Analysis and Quality Assurance, LM College of Pharmacy, Ahmedabad 380009, Gujarat, India

**Keywords:** COVID-19, SARS-CoV-2, pandemic, virus outbreak, mRNA vaccine, therapeutics

## Abstract

An unheard mobilization of resources to find SARS-CoV-2 vaccines and therapies has been sparked by the COVID-19 pandemic. Two years ago, COVID-19’s launch propelled mRNA-based technologies into the public eye. Knowledge gained from mRNA technology used to combat COVID-19 is assisting in the creation of treatments and vaccines to treat existing illnesses and may avert pandemics in the future. Exploiting the capacity of mRNA to create therapeutic proteins to impede or treat a variety of illnesses, including cancer, is the main goal of the quickly developing, highly multidisciplinary field of biomedicine. In this review, we explore the potential of mRNA as a vaccine and therapeutic using current research findings.

## 1. Introduction

Over the years, with an increase in the global population and exploitation of the environment, many diseases have emerged that have proven to be dangerous and fatal. In the case of contagious diseases, controlling the spread of disease becomes a tedious task. Therefore, to curb the spread of disease, the role of a vaccine comes into play [1,2,3]. Vaccines are designed for specific diseases so that when a person is exposed to the pathogen, the person’s body has antibodies that help protect it from the disease. Vaccination provides lasting immunity, unlike antibiotics/antivirals, which provide only temporary protection when the person is taking antibiotics/antivirals [4,5].

**Figure 1:**
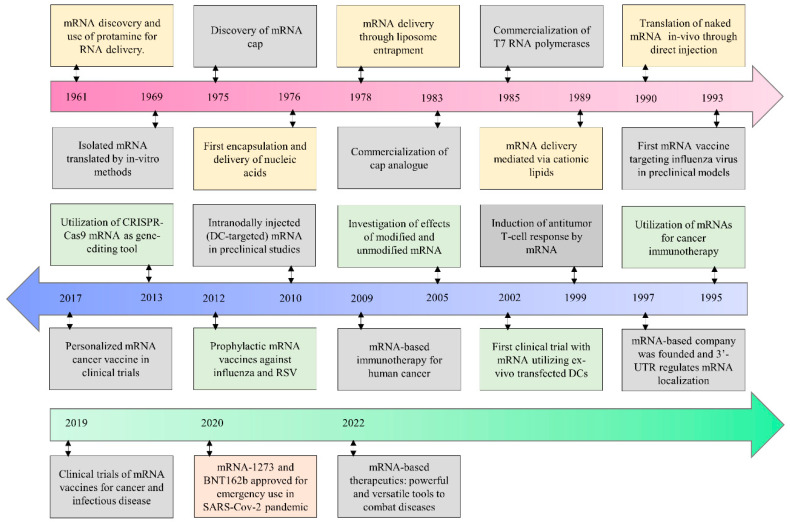
**Historical developmental milestones for mRNA-based therapeutics.** (Modified under Creative Commons Attribution 4.0 International License from [7]).

Even after the development of existing vaccines, there is a demand for new kinds of vaccine technology for viruses such as Ebola virus and Zika virus [6,7]. The developmental milestones for mRNA-based therapeutics are exhibited in Figure 1. The upsurge in virus infections and leading to epidemics of emerging strains are a source of unpredictable death rates, the quick spread of disease, and deep social impact [8,9,10]. The outbreak of coronavirus disease 2019 (COVID-19), precipitated by severe acute respiratory syndrome coronavirus 2 (SARS-CoV-2), has already alarmed the entire world with death tolls [9,11,12,13,14]. This gave rise to new vaccine technologies such as viral vector vaccines and messenger ribonucleic acid (mRNA)-based vaccines [15,16,17,18].

mRNA vaccines have provided extensively promising results in clinical trials of various deadly disorders, such as cancer [19]. Their exceptional advantages over conventional forms of medicines and vaccines, i.e., higher efficacy, fewer side effects, and easy and cost-effective production, have proven to be a breakthrough in the vaccine industry [20]. This technology also proved to be beneficial in developing a vaccine against COVID-19. These vaccines use mRNA enclosed in a lipid layer before introduction to the body. The mRNA present in the vaccine signals the immune system to generate protein similar to the spike protein present over the surface of the coronavirus, which generates an immunity barrier in the body by generating antibodies antagonistic toward the SARS-CoV-2 virus [17,21]. In this prospective work, we delve into the capabilities of mRNA as a vaccine and therapeutic with current research findings.

## 2. Current Status of mRNA Therapeutics

Some protein antigens are coded for specific nucleic-acid-based vaccines, which depend on human cells to manufacture them after injection. The manufacturing of vaccines on the basis of nucleotide sequences that will cause human cells to express the protein uses mRNA technology [22]. The identification of a possible harmless and immunogenic dose for preliminary clinical trials can be supported, in some cases, by neutralization by antibodies generated in animal models together with protection against viral exposure in vaccinated animals [23]. From a regulatory standpoint, it might be adequate to proceed with a chemistry, manufacturing, and controls (CMC) package that includes critical quality attribute (CQA)-based diagnostics for conformational purity, genetic identity, and content in the initial stages of clinical trials (phase 1/2a) [24,25]. The verification of an antigen-specific immune response in animal studies utilizing a serological assay such as the plaque reduction neutralization test may validate this. Furthermore, a potency assay in phase 3 is anticipated to show that the protein antigen is expressed in a pertinent cell line and is associated with in vivo expression. These suggestions have recently been offered by the World Health Organization (WHO) guidelines and regulatory documents from the European Medical Agency (EMA) and the United States Food and Drug Administration (USFDA) [26,27]. The Official Control Authority Batch Release (OCABR) of mRNA vaccines provides a guideline that lists appearance, identification, potency, and integrity analyses [28].

Qualitative evidence of antigen expression in transfected cells can be provided by Western blot analysis [29]. Flow cytometry can be used to quantitatively evaluate antigen expression in nucleic acid-based vaccines.

The mechanism of action is that mRNA antigens are carried directly into the cell cytoplasm, where they are translated into the appropriate protein. Additionally, plasmid DNA (pDNA), a brand-new type of vaccination, is now accessible. pDNA antigens need intracellular delivery to carry out transcription in the cell nucleus and then to transit into the cytosol, where translation takes place [30]. Generally, pDNA vaccines have a higher dose than mRNA vaccines due to their different modes of action. Hence, the doses are not generally comparable [31]. The most effective way to administer pDNA vaccinations is via electroporation using specialized equipment following intramuscular or subcutaneous injections. Whereas mRNA antigens are frequently less physically stable and need encapsulation by lipid nanoparticles (LNPs) to provide protection from RNases, pDNA antigens exhibit greater stability and simplified formulation needs. Additionally, this leads to a disproportionately higher volume of release and characterization tests for mRNA-based drug products (DPs) [32,33].

According to Dolgin E [34], “As yet, no mRNA candidate designed to replace or supplement a deficient disease-linked protein has advanced past first-in-human testing, nor has any mRNA-based product that encodes a nonnative therapeutic protein, such as a monoclonal or bispecific antibody or a Cas endonuclease for gene editing. Some of the distinct challenges of making mRNA therapeutics compared with mRNA vaccines include stabilizing the molecules for lasting therapeutic effects and maximizing potency to minimize inflammatory reactions at the injection site while still achieving high levels of protein expression. As for the encoded protein itself, it must be functional with the relevant posttranslational modifications, splicing, and expression in the right cellular location, avoiding off-target expression to preclude side effects.” The use of mRNA technology in rare genetic disorders in humans is an exciting new arena to explore. Currently, very few studies have reached human trials. Translate Bio is the first company to conduct mRNA therapy testing for cystic fibrosis in humans (NCT03375047) [35].

RNA vaccines can be divided into traditional mRNA-based vaccines and self-amplifying mRNA (SAM) vaccines. Self-replicating RNA, also known as saRNA or SAM, is a development of the mRNA substrate that can provide an immunological response at a smaller dose than is required by normal mRNA [36]. The difference between traditional mRNA vaccines and SAM vaccines is that the former contains only the target antigen gene and cannot replicate itself, while SAM encodes the engineered genome of the RNA virus, which contains the virus’ nonstructural protein gene and the antigen gene that replaces the structural protein gene. The resulting RNA can express antigenic genes at a high level due to the amplification effect of the RNA template, and these replicons cannot produce infectious virions because they delete the gene of the structural protein of the virus and cannot spread to adjacent cells [37].

**Figure 2:**
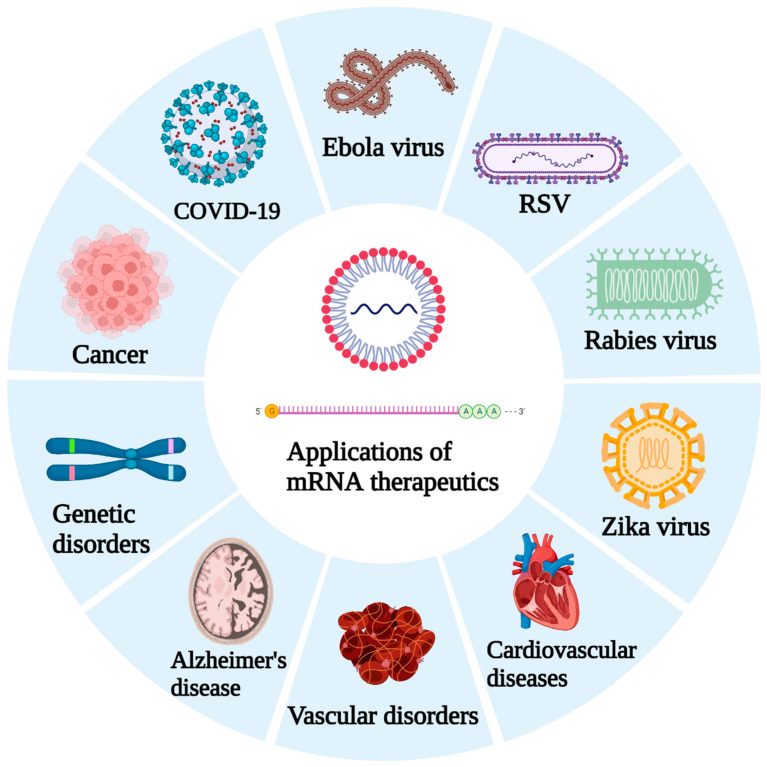
Strategies and potential application of mRNA-based therapeutics.

Replicate Bioscience is developing self-amplifying RNA (SAM) constructs for avoiding drug resistance in various cancers and for conquering autoimmune disorders. The current scenario of the development of mRNA vaccine technology in noninfectious and infectious diseases other than SARS-CoV-2 is summarized in Table 1. Developing mRNA drugs might be a complex task, but it is worthy of contemplation. The potential application of mRNA technology to combat a variety of diseases is expressed in Figure 2.

These vaccines could be used to target short interfering RNA (siRNA), cystic fibrosis transmembrane conductance regulator (CFTR), New York Esophageal Squamous Cell Carcinoma-1 (NY-ESO-1), melanoma-associated antigen 3 (MAGE-A3), and programmed cell death (PD).

### 2.1. Bioanalytical Advances in mRNA Platforms

Using an antigen-specific antibody to identify intermediate double-stranded RNA (dsRNA) and similar protein expression in individual living cells, an in vitro potency assay for a sa-mRNA complex was created to assess the replication rate [32]. The baby hamster kidney (BHK) cells used in this assay were transfected with antigen-encoded mRNA, and the primary antibody was stained with a fluorochrome. This test depends on the indirect antibody labeling technique. Within 48 h, the proportion of fluorescently positive cells revealed the degree of protein expression, verifying antigen identification. This method can be easily modified to recognize several proteins in a single cell that can be utilized as a platform-based method by identifying various antigen-specific antibodies employing secondary antibodies [30]. An indicator of RNA amplification is intracellular dsRNA produced during cell replication. Increased numbers of dsRNA-positive cells labeled by anti-dsRNA antibody were observed in BHK cells transfected with influenza and Zika virus sa-mRNA, indicating the start of self-amplification [38]. Unexpectedly, it was discovered that the immunogenic pattern of the Zika virus SAM vaccine was identical to the proportion of dsRNA-positive BHK cells and protein expression. Furthermore, electroporated cells containing RNA vaccines can potentially be used to construct direct and sandwich ELISAs. Sa-mRNA concepts are a part of the existing group of COVID-19 vaccination candidates based on mRNA [39,40].

mRNA vaccine researchers have considered antigen delivery effectiveness and stability. Because capping at the 5′-end may affect stability and translation, the proportion of encapsulated RNA is a CQA that is measured as the capping efficiency [41]. Lipid nanoparticles (LNPs) have been extensively used in therapeutic product formulation and administration to induce an innate immune response; these investigations include clinical trials [42]. Furthermore, it was revealed that mRNA transcripts synthesized in LNP and encoding for prefusion RSV F-protein induced a shielding immune response in mouse models. mRNA is often enclosed in LNP in such preparations. Encapsulation effectiveness ought to be analyzed as a CQA. The intended standard range must be maintained for the mean hydrodynamic size and size distribution (polydispersity) of LNP. Methods for analyzing such characteristics include dynamic or multiangle light scattering. It is important to keep an eye on the quantity and quality of every lipid utilized in the LNP because these factors are probably related to its efficiency and stability [43,44]. Two potential Zika virus vaccines made up of cationic nanoemulsions (CNEs) were shown to generate strong protection in nonhuman primates and mice. In this instance, the CNE adjuvant and mRNA antigen can be combined simply [45,46].

Since dsRNA encapsulated into LNP is responsible for unfavorable local injection-site immune responses, it is crucial to eliminate this from the finished product arising from in vitro-generated mRNA transcripts [6,47]. A potential Rabies glycoprotein mRNA vaccine that was freeze-dried has greatly increased thermostability in terms of the physical stability of mRNA-based vaccinations. During preservation, it is important to focus on genetic stability throughout that period [48].

### 2.2. Recent COVID-19 Vaccines from the mRNA Platform

Techniques based on mRNA are a comparatively recent framework. In December 2019, severe acute respiratory syndrome coronavirus 2 (SARS-CoV-2) intimidated global citizens as the number of cases and mortality rates escalated. As prevention is the most effective paradigm for combatting a global pandemic, the development of potential vaccines demanded a lightning-fast pace [49]. Two SARS-CoV-2 vaccines centered on modified mRNA have proceeded quickly through the clinical stages [17]. Both rely on nonreplicating mRNA sequences that code for the whole S-protein and are packaged in LNPs to guarantee safety against RNases. Although mRNA vaccines from different manufacturers utilize LMP as a drug-delivery molecule, they demand different storage conditions. The Moderna COVID-19 vaccine should be stored at −20 °C, whereas the BioNTech/Pfizer vaccine needs to be stored at −80 °C. Nevertheless, the shelf life of both vaccines is similar: 6 months. The difference in the storage conditions of both vaccines is largely because of extra precautions taken by BioNTech/Pfizer. When looking at the formulations of both vaccines, the active ingredient, vector and pH are identical, with some variation in excipients used [41,50].

**Figure 3:**
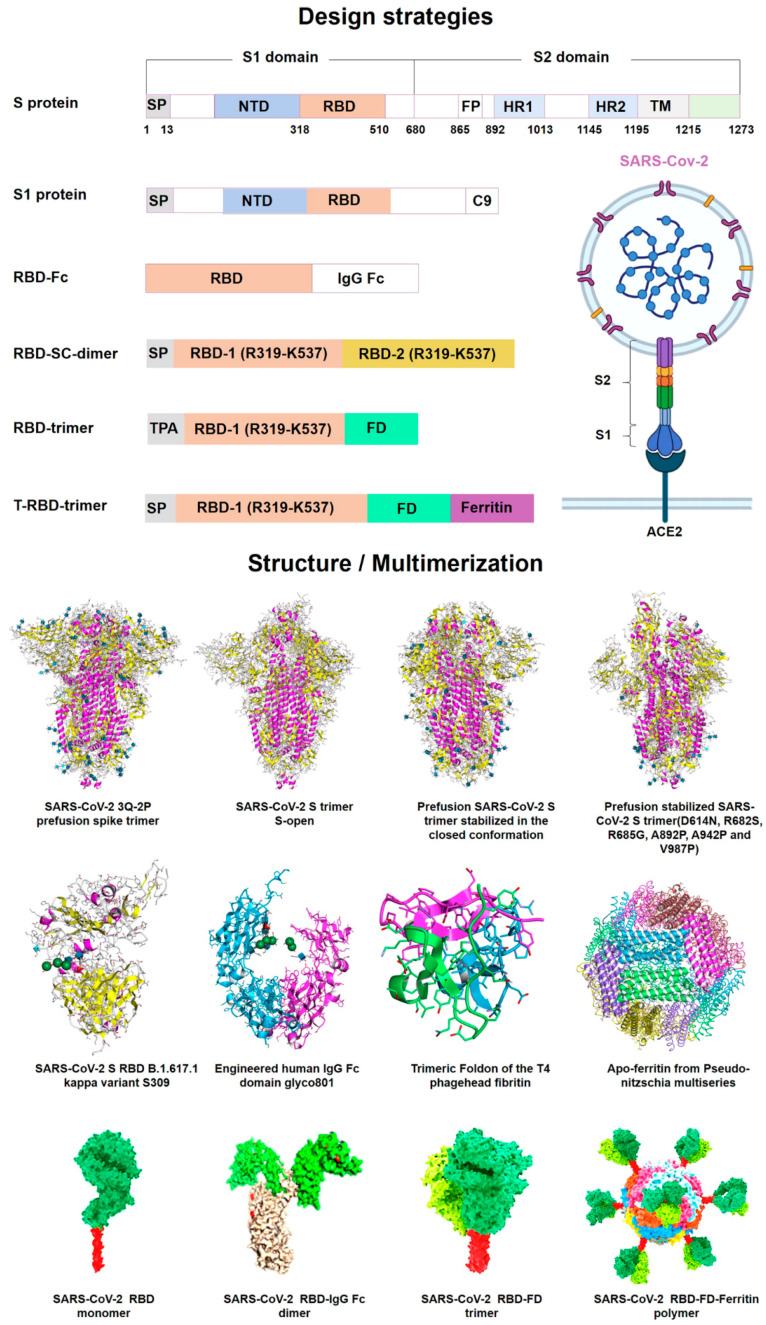
**SARS-CoV-2 mRNA antigen immunogenicity and vaccine design.** Full-length S-protein or RBD as a vaccine immunogen has been widely confirmed to induce high-affinity neutralizing antibodies. The SARS-CoV-2 S protein is intrinsically metastable and can be stabilized in a prefusion conformation by structure-based design. Prefusion-stabilized SARS-CoV-2 spike immunogen induces potent humoral and cellular immune responses. The RBD peptide is one of the most promising targets to design candidate vaccines. However, RBD has a low molecular weight, which leads to its weak immunogenicity, and can be further improved by forming multimers. Multimerization of RBD protein using humanized IgG Fc, T4 trimerization (FD), or ferritin has been shown to induce higher neutralizing antibodies compared to monomeric antigens, which will provide us with new ideas for designing powerful mRNA vaccines. (Adopted under Creative Commons Attribution 4.0 International License from [7]).

These recent developments highlighted the significance of drug-delivery systems as an essential part of the drug-development process [41]. Robust research into various delivery platforms can further improve mRNA vaccine technology. Both vaccines have shown >90% efficacy against SARS-CoV-2 and tolerable safety profiles in clinical trials. Real-world evidence after mass vaccinations has further strengthened these claims [25]. The analyses that were conducted on BNT162b2 are fully summarized in the EMA Assessment Report on BNT162b2. In vitro bioanalytical batch release along with characterization assays for the antigen and LNP are examples of product-specific tests [51,52]. Moderna and Pfizer BioNTech’s high-speed creation of COVID-19 vaccines revealed the power of this method in infectious illness—but only as a prophylactic strategy, not to cure existing illness. Recently, the fourth dose of the Pfizer BioNTech vaccine (BNT162b2) in adults older than 60 years was investigated. To date, it has shown promising results by reducing mortality after the appearance of the omicron variant [53]. Figure 3 shows the vaccine design and mRNA antigen immunogenicity in SARS-CoV-2.

## 3. Emphasis Following the COVID-19 Pandemic

During the past three years, the SARS-CoV-2 virus, with its numerous waves and different strains, has struck the whole world and has affected every aspect of people’s livelihoods, affecting many lives worldwide. The pandemic has influenced social, economic, and health-related stature globally and has also diminished the possibilities of its growth [1,54]. The lockdown and quarantine affected people’s mental well-being, and there was a loss in earning prospects that made life difficult for people. Moreover, an insufficient workforce created a lack of social protection, and disturbance in the provision of healthcare services gave rise to increasing death tolls.

The only positive side that could be seen is research and development of alternative treatments, new chemical entities, and the discovery of a vaccine [3]. The lightning-fast progress in developing a vaccine has been astounding. The generation, meticulous testing, and fabrication of multiple vaccines using different strategies have been successful due to years of outlay in extensive technology development. mRNA vaccine advancement proved to be a blessing in disguise for this pandemic. To prepare for pandemics in the future, this lesson should be generalized, as many more pathogens are lying out there in the environment to cause more lethal diseases. Existing drugs such as hydroxychloroquine were initially effective in the primitive treatment of the disease that eventually shifted focus to trials of that drug, but it lacked complete effectiveness [55,56]. This led to development in the field of treatment by convalescent plasma therapy, Molnupiravir, and many other COVID-specific drugs [57,58,59].

There was also a dire need for testing procedures that could provide rapid and reliable results, giving rise to testing features such as rapid antigen testing and RT-PCR [60]. The lesson learned from the testing strategy is to develop more stringent testing procedures so that the disease can be curbed from spreading at the point of initiation itself [61].

There were three goals to achieve during the course of the pandemic. The first was to reduce the spread of the disease, the second was to provide effective treatment and/or develop a vaccine that can generate antibodies to fight against already unfurled virus, and the third was to control the death rate [2,10,11,16,62,63,64,65,66]. Strategies to achieve these goals included implementation of lockdowns to reduce human interaction and the spread of disease, target tracing of symptomatic and asymptomatic but carrier individuals, epidemiological investigation, home isolation, and monitoring of infected persons [67,68]. This whole strategy could also be implemented in the future if any other similar kind of situation arises. This pandemic has prepared and alerted the healthcare system to have more advancements and to increase personnel trained to treat the infected individuals; to increase the facilities of healthcare, including the number of beds in individual hospitals; and to meet the demands of life-saving provisions such as oxygen gas cylinders and shortage of certain essential medicines. Additionally, the government of each country should focus more on the health budget of the country and should develop enough facilities for large population groups so that no individual has to face a lack of medical facilities and be vulnerable to death due to such conditions [69].

## 4. Carriers for mRNA Delivery

The therapeutic use of functional mRNA delivery is promising. A newly developed method of immunization and treatment is the functional delivery of RNA molecules [70]. Although naked mRNA transport has been documented, its effectiveness is constrained by nucleic acid-induced inflammatory reactions, low cellular penetration, and high rates of plasma RNA breakdown [71]. mRNA molecules are larger in size, hydrophilic, and possess a negative charge, which makes it challenging for them to enter negatively charged plasma membranes [72]. To address these constraints, RNAs have been formulated into various carrier molecules, such as lipids, polymers, peptides, exosomes, and cationic nanoemulsions.

### 4.1. Lipid-Based Delivery

Following the development of COVID-19 mRNA vaccines, three largescale developers, CureVac, Moderna, and BioNTech, have massively invested in lipid-based carriers, making them a widespread mRNA delivery system [73]. Lipids and lipid derivatives have been extensively used to formulate lipid-derived nanoparticles (LNPs) [74,75]. LNPs are nonviral carriers that are composed of phospholipids, cholesterol, polyethylene glycol (PEG), and neutral helper lipids. To prevent LNPs from being aggerated and phagocytosed by immune cells, PEG is attached to the surface of LNPs [76,77]. LNP molecules exhibit certain traits that make them remarkable carrier molecules for mRNA delivery. High mRNA encapsulation efficiency protects mRNA molecules from degradation and the capability to efficiently deliver mRNA molecules into the cytoplasm through endocytosis mechanisms [46]. These lipid molecules form convenient carriers due to their positive charge, which electrostatically interacts with the negatively charged mRNA for delivery. Owing to the lipid bilayer structure of the cell membrane, lipid-encapsulated mRNA can easily penetrate it [78,79]. The ionizable cationic lipids used in the Pfizer–BioNTech and Moderna mRNA vaccines (mRNA-1273 and BNT162b2) are SM-102 and [(4-hydroxybutyl)azanediyl]di(hexane-6,1-diyl) bis(2-hexyldecanoate) (ALC-0315), respectively [80]. Even though LNP-mRNA vaccines have shown remarkable potential, some factors impede their success, such as poor preparation reproducibility, easy oxidative degradation, and allergic reactions. Several factors are correlated with the appearance of allergic reactions: the immunogenicity of PEG and the immunogenic behavior of ionizable cationic phospholipids [43,81]. Overall, LNP molecules exhibit high delivery efficacy and excellent biocompatibility, making them a leading candidate mRNA carrier.

### 4.2. Polymer-Based Delivery

Polymeric materials provide the edge as carrier molecules due to their ability to protect mRNA from degradation and promote intracellular delivery [82]. Polymers can be classified as cationic polymers [polyamidoamine (PAMAM) dendrimer, polyethylenimine (PEI), and polysaccharide] and anionic polymers (poly D, L-lactide-co-glycolide) [83]. One such example of the utilization of cationic polymers is the mRNA molecule encoding HIV gp120 delivered by PEI formulation as intranasal vaccination in mice [84]. The limitation of polymeric materials is polydispersity, which can be tamed by incorporating lipid chains and hyperbranched groups [84]. Additionally, biodegradation-promoting domains are incorporated to facilitate the clearance of these large molecules. Overall, polymer-based delivery has exhibited promising results in preclinical studies, but further investigation is needed to gain conclusive evidence.

### 4.3. Peptide-Based Delivery

Peptide-based delivery systems exert two potential advantages as mRNA carriers. First, as peptides are highly positively charged molecules, they can efficiently bind with mRNA owing to electrostatic interactions. Additionally, peptides can protect mRNA from degradation in the serum [85]. One of the cationic peptides that can deliver mRNA is protamine. The protamine-encapsulated mRNA exhibits structural resemblance with mRNA in the nucleocapsids of the RNA virus [86]. The protamine-mRNA complex can exert a strong immune response via secretion of type-I interferon, IL-12, TNF-alpha, and activation of the TLR-7/TLR-8 pathway [87]. The hydrophilicity of protamine molecules renders it difficult to penetrate the cell membrane and evade exosomes. Exosome-destabilizing agents can be utilized to overcome such limitations [88].

### 4.4. Exosome-Mediated Delivery

Exosomes are a subgroup of extracellular vesicles that are secreted by almost all the cells in the body. They possess the potential to offer low toxicity, acceptable biocompatibility, considerable designability, and immunogenicity. They offer the advantage of delivering both hydrophobic and hydrophilic molecules [89]. mRNA incorporated into exosomes can be present as a supplier of novel proteins in beneficiary cells. One study in cocultures of glioblastoma cells indicated that mRNA transported within exosomes was translated almost less than an hour after exosome uptake [90]. Exosome-based mRNA formulations evoke enduring cellular and humoral responses to nucleocapsids and spikes, suggesting that exosome-based mRNA formulations constitute a formerly untapped platform in the battle against COVID-19 and other viral illnesses [91,92]. The large-scale production and encapsulation adaptability of exosomes are hurdles to their use as potential carriers for mRNA delivery [93].

### 4.5. Cationic Nanoemulsion (CNE)

CNE is a nonviral delivery system that amalgamates nanoemulsions along with cationic lipids. Nanoemulsions are prepared by utilizing hydrophilic and hydrophobic surfactants to stabilize the oil core in the aqueous phase to generate particles [94,95,96,97]. One such example is MF59, which is an oil-in-water nanoemulsion adjuvant that is approved by the FDA for use in an inactivated flu vaccine in adults [98]. Brito et al. investigated CNE-delivered SAM in a preclinical setting. Several vaccine antigens against human cytomegalovirus (hCMV), respiratory syncytial virus (RSV), and human immunodeficiency virus (HIV) are encoded by SAM. The results demonstrated a robust immune response at low doses [99]. Overall, CNEs have exhibited their potential for mRNA delivery in preclinical studies but await conclusive evidence from clinical trials.

## 5. Pulmonary mRNA-Based Vaccines and Therapeutics

Route of administration plays an essential role in mRNA vaccine-delivery systems. Specialized delivery from routes other than intramuscular (IM) or intravenous (IV) routes can provide numerous advantages. Intranasal (IN) or intratracheal routes are felicitous for pulmonary infections such as SARS-CoV-2 [100,101]. Intranasal vaccination offers a plethora of advantages over traditional administration of vaccines [102]. It is an improved approach to mimicking the natural way of infections.

As the nasal mucosa is the first barrier for pathogens to conquer, intranasal application can provide mucosal immunity along with systemic immunity [63]. Thus, this approach may have the potential to block the transmission of organisms, unlike available injectable vaccines, which only provide systemic immunity. Moreover, it presents the benefit of reaching lung-resident memory T-cells [103]. The major drawbacks of the traditional administration of vaccines have been determined as cold-chain maintenance and patient discomfort [104]. Production of panreactive antibodies following intranasal application can be beneficial, as the amount of COVID-19 variants has been increasing [105]. Intranasal vaccines have been developed as dry powders, which allow simple storage and transportation, which can be a crucial advantage for mass vaccination programs in developing countries [106].

The administration of intranasal vaccines is noninvasive and results in little to no discomfort. Hence, it is a feasible choice for patients who are not comfortable with needle injections. The easy accessibility of the nasal cavity also provides potential advantages. It provides an opportunity for self-administration, eliminating the need for training and expertise of healthcare professionals. This is crucial in the case of a global pandemic when rapid immunization of the population is needed [105]. Preclinical and clinical studies evaluating intranasal delivery systems elicit mucosal T-cell and IgA responses and high antibody generation that shuns COVID-19 infections in the upper as well as lower respiratory tracts [84]. A well-designed approach to vaccine formulation and inhalation devices is essential to apply this challenging administration route to large-scale vaccinations. One of the major hurdles in developing intranasal vaccines is nasal clearing, as absorption is dependent upon the residence time of the vaccine inside the nasal mucosa. Additionally, the need for specialized delivery systems for intranasal application can inflict additional costs on the formulation [107]. AdCOVID, an intranasal vaccine candidate, failed to produce an adequate immune response in phase II clinical trials (NCT04679909), putting an end to its further development. Several other IN vaccine candidates, namely, ChAdOx1, BBV154, MV-014-212 and COVI-VAC, are under investigation [105]. mRNA vaccines offer several advantages over traditional approaches, as they only consist of a specific antigen and promote a directed response [6]. mRNA is the interjacent step between DNA translation and protein synthesis in the cytoplasm. Two types of mRNA are presently studied: self-amplifying RNA and nonreplicating mRNA. Nonreplicating mRNA-based vaccines elicit a response by encoding an antigen of concern. However, self-amplifying mRNA encodes viral replication machinery along with the antigen of interest [108]. Circular RNAs (circRNAs) have gained remarkable attention in the past few years due to their involvement in a wide spectrum of diseases, such as cancers, diabetes, neurological disorders and cardiovascular diseases [109]. CircRNAs are noncoding RNA molecules lacking 5′ or 3′ ends that are formed by reverse splicing of pre-mRNAs via exons or introns. circRNAs can be further classified into natural circRNAs, which are found in various organisms, and synthetic circRNAs, which are developed by a number of enzymatic or chemical reactions [110].

**Figure 4:**
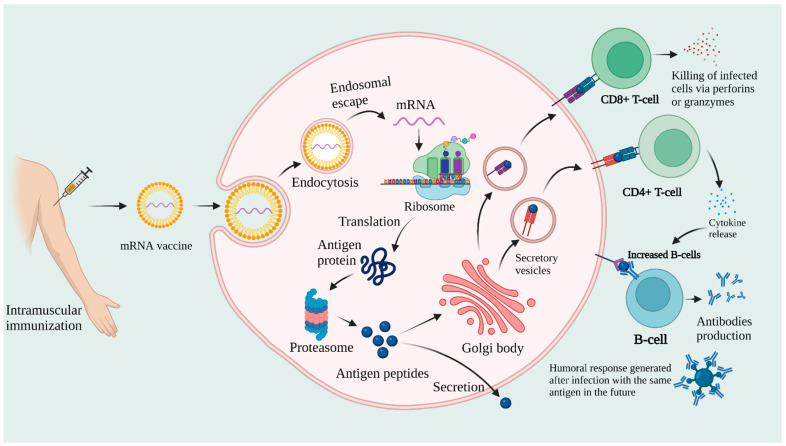
Mechanism of action of mRNA vaccines postvaccination.

Owing to their closed structure, circRNAs have the potential to accumulate in tissues and can offer a longer half-life than linear RNAs. CircRNA, which was once recognized as an error in the splicing process, has shown potential to be utilized as a novel therapeutic target and disease biomarker [111,112]. mRNA complexing with lipid nanoparticles (LNPs) is essential, as naked mRNA is rapidly degraded by RNase enzymes. Synthetic mRNA is designed to duplicate mature eukaryotic mRNA. It consists of gene sequences necessary to produce antigen protein [113,114]. Once inside the cell, the mRNA sequence is translated into the corresponding fully functional protein. This antigen protein is degraded by protease enzymes or the lysosomal pathway and bestowed on major histocompatibility complex MHC-I and MHC-II proteins to CD8+ and CD4+ T cells, respectively. The proteins can also be secreted from the cells and taken up by other antigen-presenting cells (APCs), which present them on the cell surface via MHC-II proteins to helper T cells [115]. These helper T cells stimulate B cells, which generate antibodies against that antigen. Meanwhile, memory B-cells are also generated, which provide immunity when the same pathogen is encountered in the future. The mechanism of action of mRNA vaccines is illustrated in Figure 4.

## 6. Pharmaceutical Industry Perspective

Currently, mRNA vaccines are under the spotlight in the pharmaceutical industry. They can potentially be utilized not only for infectious diseases, but also for cancer and genetic disorders [116]. Strong immunogenicity data for noninflammatory conditions are still missing. The development of mRNA vaccines for cancers (e.g., lung and breast cancer) has shown promising outcomes. One such example is BNT111, which has been confirmed to be efficacious in clinical trials for its use in melanoma [117]. mRNA vaccines are being developed to combat diverse groups of infectious diseases, such as influenza virus, Zika virus, rabies, respiratory syncytial virus, cytomegalovirus, and metapneumovirus [118]. The synthesis of mRNAs is a multistep process including in vitro reaction, purification, DNase digestion, precipitation, and filtration [119]. Readers can take a deeper dive in the detailed discussion on mRNA synthesis by Schlake et al. [119]. mRNA-1893 is a noteworthy candidate for the treatment of the Zika virus, which is currently under investigation [120]. Another candidate, mRNA-1345, is currently undergoing a phase 3 clinical trial (NCT05330975) for its use in respiratory syncytial virus when coadministered with the seasonal influenza vaccine [121]. The manufacturing of mRNA vaccines is expeditious and economical. The reason is the use of the in vivo transcription method. Thus, cloning and cell culture were omitted [122]. The pharmaceutical industry has to combat a handful of challenges in the future while improving and utilizing this technology. Appropriate formulation methods and delivery materials, when combined with the proper administration route, can increase vaccine efficacy [123]. Long-term effects of mRNA encapsulation into lipid molecules have yet to be found [124]. Novel delivery systems and lipid molecules have yet to be explored for formulation stability and better delivery. Although several approaches have been investigated to reduce cytotoxicity, such as masking cationic charges and utilizing biodegradable materials, there is a pressing need for delivery systems that offer a broad therapeutic index [125]. Humoral responses have been unimpressive when compared with the established potency of traditional live vaccines [22]. Much work is needed to achieve immunological potency without compromising the safety profile. Another intriguing realm in mRNA technology is the codelivery of mRNA vaccines. This method can provide either an enhanced immune response against one specific target, or a congregation of protein complexes [84]. As it is anticipated that the world will have to periodically battle newer pandemics, novel variants of concern may arise. Several studies suggest that mRNA vaccines may have lower efficacy for newer strains compared to the original strains [126]. Pfizer and BioNTech have recently initiated a study to evaluate the Omicron-based COVID-19 vaccine in adults aged 18–55 years. Traditional vaccines are still widely used due to their stability and high efficacy. These technologies have failed to develop immunization against many infections, such as AIDS, tuberculosis, malaria, and flu.

Shortages of raw materials and the absence of approved suppliers must be addressed. There are also some additional challenges for mRNA products. These are proprietary lipid access, supply-chain limitations, cold storage, and delivery requirements [127]. The stability of vaccines and cold-chain maintenance remain a huge challenge for mRNA vaccines. Novel approaches, such as lyophilization of mRNA-LNPs, are being investigated to tackle such concerns [123]. A minimum of 6 months of shelf life is required to cover the process of quality assurance, the release of the product, shipping, distribution, and administration to patients [128]. The pharmaceutical industry should strive to develop a vaccine that is stable, safe, efficacious, and available to all. The manufacturing process should be low-cost, reliable, and optimized to deliver on-demand supplies. The manufacturing process of mRNA vaccines is not well established, and there is scope for several improvements. New methods must be adapted to cope with market demands. Regulatory guidelines for mRNA manufacturing with special emphasis on the establishment that can deliver high-quality products are being considered.

Another major breakthrough in mRNA technology is circular RNA or circRNA. CircRNAs are RNAs in which, instead of the 5′ and 3′ open ends, the end terminals are closed covalently. These are natural (biogenic) or synthetic in nature, and the open ends are linked with the help of back splicing of exons from a single pre-mRNA. Due to its unique structural arrangement, the chances of its degradation by exonucleases are lower. CircRNAs are predominantly expressed in mammalian cells, showing cell- and tissue-specific patterns of expression. These are primarily found in the cytoplasm of a cell [109]. These are basically long noncoding RNAs. Many circRNAs have been found in different kinds of tissues and are involved in a variety of biological functions. The physiological functions of circRNA can be given as follows: miRNA sponges, As protein modulators, regulation of protein activity and As modulators. These RNAs are more stable and tend to have a longer half-life than their equivalent linear molecules. Due to their intrinsic nature, circRNAs can be synthetically generated in the form of vaccines for various protein coding and noncoding functions [129].

mRNA is a high flier in the pharmaceutical industry. This growing interest is due to the safety, flexibility, and accuracy that these technologies offer compared to traditional approaches. Multiple clinical trials from cancer therapies and genetic conditions have increased the interest of the industry to deliver these types of products into the market. Research and application of technologies that can reduce the amount of mRNA required to elicit the desired pharmacological response while increasing long-term stability, maintaining a similar safety profile, and advancing storage procedures are in demand in upcoming years [107]. Thus, overcoming the limitations and developing new production methods can revolutionize manufacturing standards and enable on-demand response during pandemics.

## 7. Quality Check and Characterization

For many years, regulatory organizations, including the WHO, EMA, and USFDA, have offered certain guidelines and recommendations for the quality control of vaccines manufactured using various technologies [130]. Maintaining control over quality and consistency is crucial in this rapidly evolving and developing environment as production is scaled up and global supply chains are developed. The necessity for sluggish and high-cost clinical bridging will be reduced in vaccine development by demonstrating analytical bridging using CQA-based (critical quality attribute-based) similarity between batches [45,131].

Assay specifications vary based on the vaccine platform and product. Potency assays are crucial for administering vaccination dosages that are both safe and immunogenic with the proper therapeutic effect. Although tests have been created for tried-and-true protocols such as LAV and recombinant proteins, some CQA can benefit from the development of quicker and more reliable in vitro tests [132].

In addition to antigens, adjuvants and excipients such as stabilizers or cryoprotectants are frequently included in the vaccine drug product’s (DP) optimized product. Key tests for these components must be included in batch release testing for DP. Additionally, any possible interaction of these components in antigen testing, such as potency, needs to be disregarded or resolved [133].

Recently, there has been a significant rise in the number of novel technologies applied to track various cell-culture-related factors to ensure quality. For instance, several techniques, including multiplex polymerase chain reaction (PCR) and STR mapping, are now offered for quick recognition of defects and quality concerns with cell lines. These techniques are used to detect contamination and misidentification of cell lines. Specifically, the recently developed techniques for the quick detection of exogenous contamination (such as the Touchdown PCR detection method for mycoplasma) not only ensure the safety of the vaccine but also address the issue of a lack of cell diagnostic techniques, which could save a huge amount of time for vaccine R&D while offering solid support for the high quality of the manufacturing process during technical evaluation [134].

Additionally, the quality of the mRNA bulk and the potency and efficacy of the vaccine also heavily depend on the quality of the nanoparticle carrier if used in the formulation, which should be carefully tuned. The stages involved in the quality checks of the high-volume mRNA targets for mRNA identification, mRNA sequence length, sequence accuracy, and integrity [48]. The optimization of particle size, polydispersity index (PDI), encapsulation rate, stability, surface charge, immunogenicity, and other properties of a nanoparticulate carrier should be the focus of research aimed at improving their quality. Both in vitro and in vivo testing should be used to determine the therapeutic activity of the manufactured vaccine [135].

Additionally, the WHO has recently published a general guideline for RNA vaccines that outlines important specifications for vaccine design. Clarifications should be made regarding the selection of the target antigen(s), encoded proteins (such as cytokines), the inflammatory nature of the given mRNA, and the quality, amount, and bias of the immune responses (such as the T helper (Th1/2) cell phenotype), and biostability [136].

## 8. Radical Frameshift

In the last two centuries, vaccines have remained at the forefront as prophylaxis against infectious diseases. Prophylactic vaccination offers preexposure protection, as well as the evolution of herd immunity. Therapeutic vaccination-boosted immunomodulation also allows the treatment of cancers and infectious diseases. The established clinical value of licensed vaccines encourages additional research and development of newer vaccination advances. These aim to improve prophylactic and therapeutic efficacy, develop advanced technologies, streamline manufacturing processes and enable a swift response to emerging infectious, genetic and lifestyle diseases. Nucleic-acid-based immunotherapy exemplifies one such method, where synthetic sequences are utilized for in situ expression of antigenic proteins or peptides [137].

During fast-track COVID-19 vaccine development, messenger RNA (mRNA) has become known as a genetic platform technology for the advancement of vaccines not only against infectious diseases, but also for oncological applications. mRNA transforms cells into antigen production biofactories as in situ antigenic vaccines that trigger potent cellular and humoral immune responses [133].

In contrast with protein-based vaccines, mRNA’s specific biochemical and biophysical properties allow for simple multiplexing of vaccine antigens without formulation issues. The primary benefit of RNA vaccines compared to DNA vaccines is that they directly exert their function in the cytoplasm without requiring transport mechanisms or cellular transcription machinery. Furthermore, RNA has an extremely short half-life in the body and cannot integrate into the host genome, which contributes to a good indication of its safety profile. Additionally, the limited expression capabilities of conventional mRNA vaccines can be overcome by self-amplifying mRNA (SAM) vaccines whenever a higher level of immunomodulation is needed. SAM vaccines, which are genetically engineered into the element of an RNA virus genome, allow magnification of the antigen-coding region, resulting in higher levels of expression and therefore a smaller dose required with the SAM vaccine.

Another way forward with mRNA therapies is the newer route of administration, as current vaccines are limited to i.m. and i.v. routes. However, promising results were achieved in addressing different delivery modes for patient convenience. Recently, an intranasal spray of mRNA COVID-19 vaccine candidate was investigated. In another study, a milli-injector capsule with an mRNA nanoparticulate vaccine was delivered orally to the gastric mucosa [138]. Furthermore, SARS-CoV-2 and norovirus sequences have been mixed to produce an orally administered SAM vaccine. New mRNA vaccine development must address challenges such as long-term stability and scalability to meet the world’s needs. Novel lipid-based nanoformulations may require further adjustment in lipidic composition and positive charge ratio to lower or no adverse effects and biodegradability [25]. mRNA- and SAM-based immunological vaccines, in particular, face the hurdles and challenges of development and GMP level scale-up, clinical trials, and subsequent regulatory approval. These challenges also include mRNA stability and designing an effective delivery formulation. These hurdles can be overcome by adopting a methodical and risk-based approach. Tackling these issues is vital to break the ground for more mRNA vaccines to enter the healthcare market.

## 9. Conclusions

Research on mRNA vaccines has been ongoing for decades, but it was not until the COVID-19 pandemic that this technology gained remarkable attention. For the past 2 years, there has been a surge of preclinical as well as clinical evidence reporting the effectiveness and safety of mRNA-based vaccines. The use of mRNA technology is no longer limited to the COVID-19 pandemic. Recent evidence has shown the potential of mRNA vaccines to counteract a wide range of infectious pathogens and cancer. mRNA vaccines are safe, efficacious, and easy to manufacture on a large scale. These characteristics become essential when combatting global pandemics in the future. As with every eminent therapeutic platform, mRNA vaccines have their shortcomings. Adverse effects should be addressed by introducing biocompatible lipids and targeted delivery of vaccines into dendritic cells. Exploring other routes of administration, such as intranasal delivery and milli-injector capsules, can offer various advantages over traditional approaches. In the case of specific disease targets that may require a longer half-life, self-amplifying mRNA technology can provide numerous advantages. However, the establishment of well-equipped continuous manufacturing capabilities is yet to be established worldwide. Thorough guidelines for manufacturing mRNA vaccines can be expected in the future. In summary, mRNA technology possesses the potential to unveil a new era of medicine. Extensive improvements in the current vaccination approaches and leading-edge research on mRNA-based therapeutics can be expected in years to come.

## Figures and Tables

**Table 1:** Current scenario of the development of mRNA vaccine technology.

Disease	Drug Molecule	Target	Remarks	Reference
Development of mRNa technology as a therapeutic target				
Cystic fibrosis	MRT5005	CFTR gene	Upon inhalation, MRT5005 delivers the mRNA molecules that encode for functional CFTR protein directly into bronchial epithelial cells. In the interim data analysis of phase-I/II, the molecule was found to be safe and tolerable with no serious side effects.	NCT03375047
Melanoma	BNT111	TAAs (NY-ESO-1, MAGE-A3, tyrosinase, and TPTE)	The molecule is currently in phase-II clinical trials for its use in patients with anti-PD-1-refractory/relapsed unresectable Stage III or IV melanoma in combination with Cemiplimab. Phase-I results have confirmed the safety and dose of this molecule. Some preliminary antitumor responses were also repoted.	NCT04526899
Development of mRNA technology in infectious diseases other than SARS-Cov-2				
Zika virus	mRNA-1893	-	The molecule is currently under phase-II clinical trials; phase-I results exhibited clear neutralizing antibody response.	NCT04917861
Respiratory syncytial virus	mRNA-1345	Prefusion F glycoprotein	The vaccine is to be administered in adults 60 years and older, along with a seasonal influenza vaccine.	NCT05330975
Rabies	CV7202	Rabies virus glycoprotein	In the phase-I study, the vaccines were found to be well tolerated and exhibited adequate neutralizing antibody responses.	NCT03713086
Chikungunya	mRNA-1944	Anti-CK virus mAb	mRNA-1944 is mRNA coated monoclonal antibody CHKV-24. This phase-I trial reported an encouraging safety profile and detectable neutralizing activity.	NCT03829384

## Data Availability

Not applicable.
